# Switchable Solvent for Separation and Extraction of Lignin from Lignocellulose Biomass: An Investigation of Chemical Structure and Molecular Weight

**DOI:** 10.3390/polym16243560

**Published:** 2024-12-20

**Authors:** Debao Li, Letian Qi, Magdi E. Gibril, Yu Xue, Guihua Yang, Mengru Yang, Yujie Gu, Jiachuan Chen

**Affiliations:** 1 State Key Laboratory of Biobased Material and Green Papermaking, Qilu University of Technology (Shandong Academy of Sciences), Jinan 250353, China; 10431221053@stu.qlu.edu.cn (D.L.); magdigibril@qlu.edu.cn (M.E.G.); xxyy0707@163.com (Y.X.); 10431230284@stu.qlu.edu.cn (M.Y.); 10431230283@stu.qlu.edu.cn (Y.G.); chenjc@qlu.edu.cn (J.C.); 2 Faculty of Industries Engineering and Technology, University of Gezira, Wad Medani 2667, Sudan

**Keywords:** switchable solvent, lignin, lignocellulose, poplar wood, extraction

## Abstract

Lignin, the most abundant natural aromatic polymer, holds considerable promise for applications in various industries. The primary obstacle to the valorization of lignin into useful materials is its low molecular weight and diminished chemical reactivity, attributable to its intricate structure. This study aimed to treat lignocellulosic biomass using a switchable solvent (DBU–HexOH/H_2_O) derived from the non-nucleophilic superbase 1,8-diazabicyclo [5.4.0]undec-7-ene (DBU), which efficiently separates and extracts lignin from poplar wood. Additionally, it sought to characterize fundamental properties of the extracted switchable solvent lignin (SSL) and propose a mechanism for its separation. In comparison to milled wood lignin, SSL exhibits a greater molecular weight, superior homogeneity, and enhanced stability. The SSL sample was analyzed using spectroscopies including infrared spectroscopy, nuclear magnetic resonance, and X-ray photoelectron spectroscopy. The findings indicated that the structure of SSL was preserved, with the switchable solvent primarily cleaving the C–C and α-O-4 bonds, resulting in a low hydroxyl content, an elevated H/C ratio, and a reduced O/C ratio. The SSL was successfully prepared to lignin nanoparticles (LNPs) with size range of 531–955 nm. This paper presents a technique for processing lignocellulosic biomass using a switchable solvent, highlighting advancements in lignin’s structure and enhancing its use in the chemical sector.

## 1. Introduction

Lignin is one major component of lignocellulosic materials, alongside cellulose and hemicellulose. It is an aromatic biopolymer consisting of guaiacyl (G), syringyl (S), and p-hydroxyphenyl (H) units, linked by various ether bonds (C–O–C) and carbon–carbon bonds (C-C), including β-5, β-β, 5-5, and β-1 links [1,2]. Lignin has undergone modifications to enable its application in industrial chemicals [3], including adsorbents, binders, dispersants and surfactants. It has also been employed in biomedicine to regulate bone metabolism and its anti-inflammatory properties [4]. Additionally, it has been utilized to produce an innovative bioplastic [5], energy storage material [6], and food preservation [7]. However, the lignin applied for high-value applications only accounts for 5% of its total productions [8,9].

Owing to its complexity, heterogeneity, and robust structure, lignin is challenging to efficiently extract from lignocellulosic resources while maintaining high-purity and remaining structurally intact [10]. This is mainly caused by the primary and secondary bonds forming between lignin and carbohydrate components [11,12,13]. The conventional approaches to lignin isolation typically entail rigorous conditions, potentially causing unwarranted breakdown and alteration of its primary constituents [14,15].

The common methods for extracting lignin include organic solvents, acids, and alkalis treatment [16]. Organic solvent treatment utilizes its strong solvation properties to disrupt the intermolecular and intramolecular forces within lignin macromolecules, facilitating the dissolution of lignin [17]. However, this method is expensive, has low separation efficiency, and can be toxic [18]. Acidic treatment breaks the aromatic ether bond of lignin, particularly the β-O-4 bond, resulting in acid-insoluble lignin residues [19]. Unfortunately, this variety of lignin experiences a condensation process, resulting in the creation of a stable polymer, which prevents further utilization [20]. Similarly, the bonds that bind lignin to carbohydrates are easily broken when lignin is extracted by alkalis treatment. Unfortunately, the nucleophilic reagents alkaline environment, like OH^−^, can cleave the numerous ether linkages within lignin, including α-aryl ether, non-phenolic β-alkyl ether, and phenolic β-aryl ether bonds [17], which facilitates the breakdown and dissolution of lignin macromolecules and lowering molecular weight (MW) as a consequence [21,22]. Additionally, alkalis treatment entails several shortcomings, including the non-recyclability of alkali additives, high energy usage, and environmental contamination [23].

Lignin has been recently extracted and separated from lignocellulosic biomass through the use of ionic liquids (ILs) synthesized from the non-nucleophilic superbase 1,8-diazabicyclo[5.4.0]undec-7-ene (DBU). The complex structure of lignocellulose biomass, along with the hydrogen bonds and π–π interactions between the ILs, significantly influences the treatment procedure [24,25]. Switchable solvent, formed by mixing DBU and hexanol (HexOH), passing a typical trigger of acid gas CO_2_, presents unique “switchable” chemical and physical properties such as hydrophilicity, polarity, ionic strength properties, dielectric constant, and hydrogen-bonding ability [26,27]. Utilizing this characteristic, the switchable solvent can be applied to the extraction process, followed by adding or removing the trigger (CO_2_) to adjust its physical chemistry properties [28]. There has been research on the application of switchable solvents for the separation of cellulose [29] and hemicellulose [30] from lignocellulosic biomass. Recently, Qi’s group [31] announced the creation of a switchable solvent composed of DBU, HexOH, and deionized water (DI), demonstrating significant lignin solubility while exhibiting negligible cellulose solubility. However, as far as we know, there is no information on how the switchable solvent (DBU–HexOH/H_2_O) influences the chemical structure and MW of the extracted lignin. As a result, the purpose of this research was to look into the possibilities of switchable solvents for lignin extraction and examine how they affect the quality, chemical structure, and MW of the extracted switchable solvent lignin (SSL).

This study proposes a switchable solvent system based on N-heterocycles (DBU–HexOH/H_2_O) for the targeted extraction of lignin from poplar wood lignocellulosic biomass. The extracted SSL was assessed for MW and chemical structure through elemental analysis, gel permeation chromatography (GPC, e2695, Agilent Technologies Inc., Palo Alto, CA, USA), and thermal gravimetric analysis (TGA, SDT650, TA Instruments, Milford, MA, USA). The bond-breaking process of SSL samples was investigated using Fourier transform infrared spectroscopy (FT-IR, BRUKER ALPHA, Ettlingen, Germany), two-dimensional heteronuclear single quantum coherence nuclear magnetic resonance (2D HSQC NMR, BRUKER AVANCE III 500 MHz, BRUKER, Karlsruhe, Germany), ^31^P NMR, and X-ray photoelectron spectroscopy (XPS, ESCALABXi+, ThermoFisher Scientific, London, UK). To evaluate the efficiency, chemical structure, and MW, the obtained findings were contrasted with milled wood lignin (MWL). Scanning electron microscopy (SEM, Regulus 8100, Hitachi Scientific Instruments (Beijing) Ltd., Beijing, China) and zeta potential and nanoparticle sizing tester (Zetasizer Nano ZS90, Malvern Panalytical, Malvern, UK) offered understanding of the physical shape and size distribution of the prepared lignin nanoparticles (LNPs).

## 2. Materials and Methods

### 2.1. Materials

Poplar wood chips, sourced from a pulp mill in Shandong, China, with compositions of 46.40% cellulose, 31.48% hemicellulose, 21.56% lignin, 2.65% alcohol-benzene extractive, and 0.32% ash were used. The following chemicals were acquired from the Shanghai Macklin Biochemical Technology Co., Ltd. (Shanghai, China): 1,8-diazabicyclo[5.4.0]undec-7-ene (DBU, analytical grade, 99%), γ-Valerolactone (GVL, 98%), 1,4-dioxane (analytical grade, 99%), tetrahydrofuran (THF, ≥99.9%), dimethyl sulfoxide-d_6_ (DMSO-d_6_, 99.9%), cyclohexanol (gas chromatography grade, >99.0%), chloroform-d (CDCl_3_, 99.8%), chromium(III) acetylacetonate (98%), and potassium bromide (KBr, spectrally pure). Ethanol (analytical grade, ≥95.0%) and acetic anhydride (analytical grade, ≥98.5%) were supplied by Sinopharm Chemical Reagent Co., Ltd. (Shanghai, China). Pyridine (analytical grade, ≥99.5%) and hexanol (HexOH, analytical grade, ≥99.0%) were sourced from the Tianjin Damao Chemical Reagent Factory (Tianjin, China). Acetone (analytical grade, ≥99.5%) was procured from Yantai Far East Fine Chemical Co., Ltd. (Yantai, China). The 2-Chloro-4,4,5,5-tetramethyl-1,3,2-dioxaphospholane (TMDP, 95%) was purchased from Sigma-Aldrich (Shanghai) Trading Co., Ltd. (Shanghai, China). The chemicals and reagents were analytical grade and used directly.

### 2.2. Preparation of Switchable Solvent

The synthesis of the switchable solvent followed the protocol outlined in the referenced literature [32], which involved mixing DBU and HexOH at a 1:1 molar ratio and stirring at 25 °C to produce a clear and homogeneous DBU–HexOH solvent. Subsequently, DI was incorporated into the mixture at a molar ratio of 1:5, agitated for 30 min, and gas CO_2_ was added for 0.5 h until the viscosity of the solution markedly increased.

### 2.3. Lignin Extract with Switchable Solvent

3 g of poplar wood (80~40 mesh) were mixed with 30 g of the switchable solvent, and the blend was processed at 150 °C for a duration of 6 h at 300 rpm. After reacting, the produced mixture was left to cool naturally and filtered. Acetone as anti-solvent (10 times the volume of the filtrate) was mixed with the filtrate to induce lignin precipitation. The precipitated lignin was desiccated at 105 °C for a day and named as the SSL. The solid residues were also desiccated at 105 °C for a day.

### 2.4. Preparation of Lignin Nanoparticles

According to the previous reporting methods [33], dissolve 10 mg of the SSL sample in 20 mL of 98% GVL solvent to form a clear lignin solution. Subsequently, it was placed in a dialysis bag (MW cutoff = 6000~8000 Da, JielePu, Changsha, China) and transferred to a container with DI for dialysis. The DI was replaced every 12 h for 72 h. After the end of dialysis, LNPs were collected by freeze-drying.

### 2.5. Chemical Composition Analysis

The makeup of the poplar wood before and after treatment was assessed according to the NREL analytical procedure [34]. The SSL sample (0.3 g) was loaded into a pressure bottle with 3 mL 72% H_2_SO_4_ and reacted in an oil bath at 30 °C for 1 h. Afterwards, 84 mL of DI was introduced, and the process was conducted at a temperature of 121 °C for 1 h. After the reaction was conducted, the acid-insoluble lignin was filtered and weighed after vacuum drying [35]. The ash content was determined via incinerating 3 g of lignin in a muffle furnace at a temperature of 575 ± 25 °C for 4 h [36].

The solid rate after switchable solvent treatment was calculated as the following equations:

(1) $$Solid\;residue\;rate\left(\%\right)=\frac{m_{1}}{m_{0}}\times 100\%$$

With m_0_ representing the mass of the poplar wood and m_1_ denoting the mass of the solid residue.

The lignin removal was determined using the following equations:

(2) $$Lignin\;removal\;rate\left(\%\right)=\frac{\left(1-m_{1}\times P\right)}{m_{0}}\times 100\%$$

where P is the content of lignin in the solid residue.

The purity of SSL sample was calculated as follows:

(3) $$Purity\;of\;SSL\left(\%\right)=\frac{m_{2}+m_{3}}{m_{4}}\times 100\%$$

With m_2_ representing the mass of acid-insoluble lignin, m_3_ denoting the mass of acid-soluble lignin, and m_4_ indicating the mass of SSL.

### 2.6. Characterization of the Lignin

The 40 mg lignin was added into a mixture of 1 mL of pyridine and 1 mL of acetic anhydride solution. The mixture was then allowed to react for 48 h at room temperature. The acetylated lignin was caused to precipitate by introducing 10 mL of DI to the solution. The precipitate was washed with DI until the filtrate had no acidic odor, and then the solution was freeze-dried to yield acetylated lignin [37]. Then, 1 mg sample of acetylated lignin was dissolved in 1 mL of THF and passed through a 0.22 μm syringe filter before undergoing MW determination via GPC (e2695, Agilent Technologies Inc., Palo Alto, CA, USA). Calibration of the GPC standard curve was performed using polystyrene [38].

The stability of lignin samples under thermal conditions was assessed through TGA (SDT650, TA Instruments, Milford, MA, USA) [39]. About 8 mg of lignin were loaded into an alumina crucible. The temperature was elevated from ambient to 800 °C, with a heating rate of 10 °C/min. The crucible was flushed with high-purity N_2_ at a rate of 40 mL/min.

The lignin’s structure and chemical groups were analyzed by FT-IR spectroscopy (BRUKER ALPHA, Ettlingen, Germany). The lignin samples were mixed with KBr in a 1:100 mass ratio, then grinded and mixed well to be pressed into thin flakes. The scan was performed in the wavelength range of 4000–400 cm^−1^, with a resolution of 4 cm^−1^ and 32 scans [40].

An elemental analyzer (Elementar UNICUBE, Elementar Trading (Shanghai) Co., Ltd., Shanghai, China) was used to perform the elemental analysis on the lignin sample. The contents of C, H, O and N elements in the lignin sample were determined through the process of intense heat combustion [41].

The 2D HSQC NMR spectroscopy (BRUKER AVANCE III 500 MHz, BRUKER, Karlsruhe, Germany) was employed to examine the sub-structural types and linkages connections within lignin samples [42,43]. A 50 mg sample of lignin was accurately weighed and dissolved in 0.5 mL of DMSO-d_6_ by shaking until fully dissolved. Subsequently, the resulting solution was poured into an NMR tube, ready for analysis using an NMR spectrometer (BRUKER AVANCE III 500 MHz, BRUKER, Karlsruhe, Germany). The structure of lignin was determined by ^31^P NMR. Anhydrous pyridine and deuterated chloroform were mixed at a ratio of 1.6:1 (*v*/*v*) to configure deuterated reagents. 54.25 mg cyclohexanol was diluted to 5 mL with deuterated reagent to obtain 10.85 mg/mL internal standard solution. 25 mg chromium (III) acetylacetonate was diluted to 5 mL with deuterium reagent to obtain a relaxant of 5 mg/mL. Lignin (20 mg) was dissolved in 500 μL of a deuterium reagent, to which 100 μL of a relaxation agent and 100 μL of an internal standard solution were subsequently added. The solution was completed with the addition of 100 μL of TMDP 15 min prior to testing, after which it was poured into the NMR tube for analysis.

According to the method recorded in the literature [44], XPS analysis (ESCALABXi+, ThermoFisher Scientific, London, UK) was conducted to investigate the elemental makeup and proportions in lignin. The dried lignin samples were wrapped with aluminum foil for XPS analysis.

### 2.7. Characterization of Lignin Nanomaterials

The surface contour of the lignin nanomaterials was examined by field emission SEM (Regulus 8100, Hitachi Scientific Instruments (Beijing) Ltd., Beijing, China) [45]. The size distribution and Zeta Potential (ζ) of lignin particles were investigated using a zeta potential and nanoparticle sizing tester (Zetasizer Nano ZS90, Malvern Panalytical, Malvern, UK) [46]. The LNPs were diluted in DI (0.1 mg/mL) and then placed in the cuvette. Particle size and ζ was analyzed in the Malvern potential sample pool, the measurement was conducted three times, with the mean value recorded.

## 3. Results and Discussion

### 3.1. Poplar Lignin Extracted by Switchable Solvent

**Figure 1:**
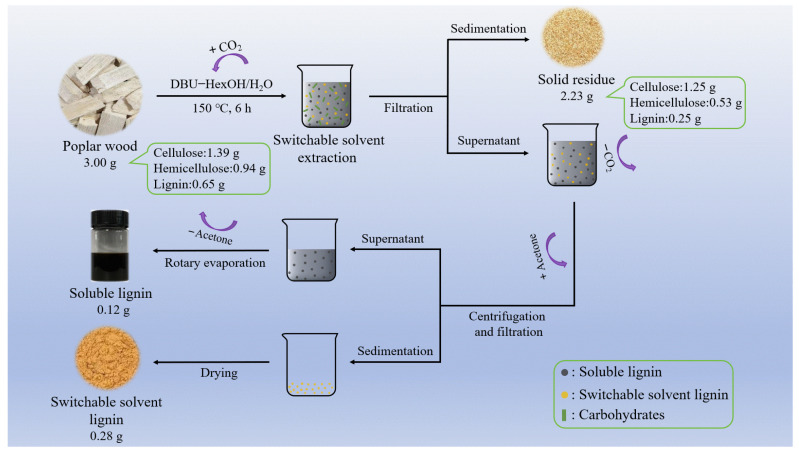
Schematic representation of lignin extraction from poplar wood using switchable solvent.

Lignin was extracted by using the method illustrated in Figure 1, which is similar to the conventional deep eutectic solvent (DES)–lignin separation and extraction method [47,48]. A switchable solvent was obtained by mixing DBU and HexOH, followed by adding DI, and finally bulging CO_2_ until the viscosity of the solution increased significantly. The required amount of poplar wood powder was added to the switchable solvent, the mixture was then exposed to a high-temperature process (150 °C, 6 h). The solid residue and the switchable solvent–lignin mixture were separated by filtration. At the same time, gas CO_2_ in the switchable solvent–lignin mixture was removed, so as to reduce the solubility of lignin facilitating the separation [28,31]. Acetone (10 times the volume) was added to the liquid phase as an anti-solvent, and the precipitate was separated by filtration at room temperature. The SSL was obtained by drying the precipitate at 105 °C for 24 h. Acetone in the supernatant was removed extracts, and response surface methodology was utilized to refine the treatment conditions (Figure S1), in which the optimized operation condition for lignin extraction was 150 °C and 6 h with the lignin removal rate of 60.87% and the yield of 55.10%. The solid residue following switchable solvent treatment contained 56.16% cellulose, 23.94% hemicellulose, and 11.21% lignin. As shown in Figure 1, lignin is selectively extracted from poplar, leaving the cellulose in the solid residue, thereby it is with high cellulose content.

The number-averaged molecular weight (M_n_), weight-averaged molecular weight (M_w_), and molecular dispersity (Đ) of the MWL and SSL samples were ascertained via GPC, with the findings detailed in Table S1. The M_n_, M_w_, and Đ of the SSL sample were 4400 g/mol, 8000 g/mol, and 1.82, respectively. Compared with the MWL, ChCl-Lac lignin [49], and LigAc lignin [50], the SSL sample presented lower Đ, indicating a more uniform molecular dispersity. The increase in M_w_ and M_n_ may be attributed to the reduction in the lignin degradation reaction in the DBU–HexOH/H_2_O treatment system. This allows the lignin to maintain a higher MW because it is tailored through hydrolyzation into smaller fragments instead of vast degradation [51]. Therefore, the lignin is extracted through switchable solvent treatment presented much higher MW than that from traditional DES treatment.

**Figure 2:**
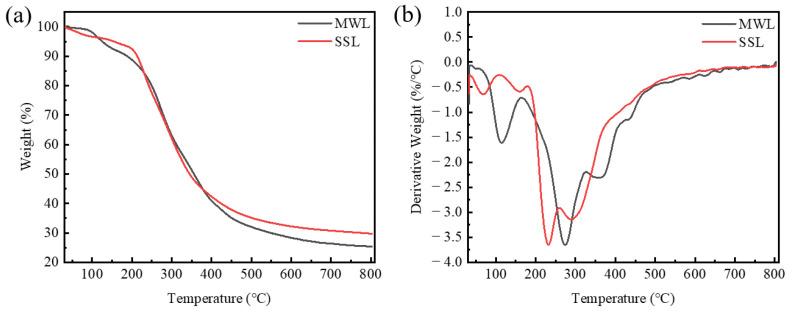
Thermal degradation profiles of lignin samples: (**a**) TGA curves and (**b**) DTG curves.

TGA of the MWL and SSL samples was performed, and the thermal stabilities of the two different lignin were compared. Figure 2a,b illustrates that with rising temperature, as the temperature increased, the TGA curve depicted a progressive mass loss for lignin. Concurrently, the DTG curve revealed an initial upsurge followed by a downturn in the weight loss rate for the lignin samples. The mass decrease near 100 °C was attributed to the vaporization of moisture present in the lignin sample. The main thermal degradation region for the lignin fractions occurred between 200 and 500 °C. The degradation occurring between 200 and 400 °C is believed to be associated with the breaking of β-O-4 and α-O-4 ether linkages, in addition to the carboxylation or carbonylation of the aliphatic hydroxyl (–OH) groups present in lignin [52]. The second stage of lignin degradation predominantly took place at temperatures exceeding 400 °C, during which the C–C linkages and methoxy (–OCH_3_) groups in lignin fragments were broken. It was evident that the SSL sample exhibited a higher carbon residue at 600 °C than the MWL sample, which was associated with its higher MW [53]. Above 500 °C, the lignin weight loss rate started to diminish gradually. This effect was likely due to the condensation reactions occurring in lignin at high temperatures, resulting in the formation of heat-resistant macromolecular compounds [38]. The residual carbon rates of the MWL and SSL samples were 25.37% and 29.76%, respectively. Generally, the carbon yield from lignin is directly proportional to the quantity of C–C linkages, presence of condensation structures, and number of –OCH_3_ [54]. The SSL sample exhibited a higher residual carbon yield compared to the MWL sample, indicating its superior thermal stability. As shown in Figure 2b, the DTG curves also include three stages. The initial weight loss observed around 180 °C in the SSL sample was ascribed to the vaporization of low MW substances. The second phase was attributed to the breakdown of aryl ethers in the range of approximately 150–260 °C, with the peak decomposition rate occurring at 231.7 °C. The third stage took place between 260 and 500 °C, which was linked to the breakdown of phenylcoumaran structures and –OCH_3_ [55]. The primary distinction between SSL and MWL was evident within the temperature spectrum of 200–400 °C, which points to the likelihood that the distribution of ether bonds is disrupted and that the side-chain fragments could be modified.

### 3.2. Structural Characterization of Switchable Solvent Lignin

**Figure 3:**
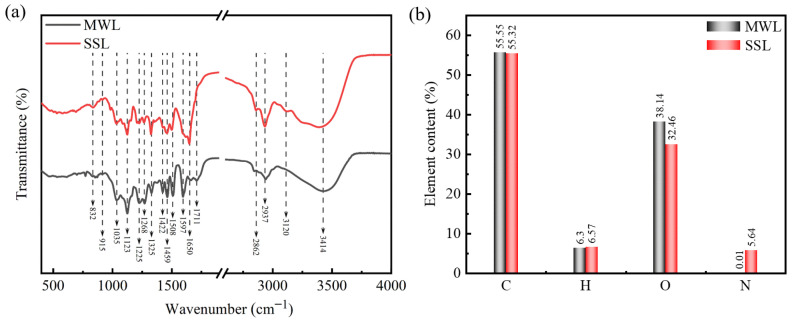
FT-IR (**a**) and elemental analysis (**b**) of lignin samples.

Structural characterization of the MWL and SSL samples were performed using FT-IR and the corresponding peak assignments detailed in Table S2. As shown in Figure 3a, the predominant functional groups in lignin are –OH groups, both aliphatic and phenolic, with the –OH stretch found at 3414 cm^−1^ [56]. The signals at 2937 and 2862 cm^−1^ are assignable to the stretching vibrations of –OCH_3_ and methylene (–OCH_2_–) groups, respectively, exhibiting significant intensity in both the MWL and SSL specimens. Studies have shown that a higher extent of methylation in lignin contributes positively to its antioxidant functionality [57]. The peak at 1711 cm^−1^ is identified as the C=O stretch vibration associated with the benzene ring. The peak corresponding to the unconjugated carbonyl group at 1711 cm^−1^ showed a decrease in intensity following switchable solvent treatment, while the strong and broad peak at 1620 cm^−1^, associated with the stretching vibrations of both the conjugated carbonyl groups and the C–C bonds in the aromatic skeletal framework experienced an increase in intensity [58]. The additional peak observed at 1073 cm^−1^ was assigned to the vibrational deformations of C–O bonds found in secondary alcohols and aliphatic ethers [59,60] and compared to the MWL, the SSL sample demonstrates an absorption peak at this location. The distinct peaks at 1597, 1508, and 1422 cm^−1^ correspond to the vibrational deformations of the aromatic rings in the phenylpropane structure. Additionally, the absorption band at 1458 cm^−1^ is a result of C–H bending vibrations within the –OCH_3_ and –OCH_2_– groups, accompanied by the vibration of aromatic rings. Besides that, the absorption peak detected at 1650 cm^−1^ signified the vibrational deformations associated with the ring-conjugated carbonyl groups within the SSL sample. The peak at 1625 cm^−1^ is the result of the Fermi resonance of the deformation vibration of –NH_2_ [61]. G units were observed at 1035 and 915 cm^−1^, whereas S units were distinctly apparent at 1325 and 832 cm^−1^. The presence of these signals confirmed the typical G and S units within poplar lignin. Additionally, the absorbance peaks for the S units were more pronounced than those for the G units, signifying a higher abundance of S-type lignin relative to G-type lignin in poplar [62]. Therefore, the FT-IR spectra proved that the spectra of the MWL and SSL samples were alike, indicating that the basic lignin structure remained intact.

Figure 3b shows that the C, H, O, and N contents in the MWL sample were 55.55%, 6.30%, 38.14%, and 0.01%, respectively, while in the SSL sample the contents were 55.32%, 6.57%, 32.46%, and 5.64%, respectively. The elemental profile of the SSL sample exhibited parity with that of the MWL sample, indicating that the lignin structure extracted by the switchable solvent was consistent with that of the MWL structure, which corroborates the FT-IR analysis outcomes. The carbon content of the two samples was quite comparable. This indicates that the fundamental carbon framework of lignin remained rather intact. Furthermore, the percent H/C of the SSL samples increased slightly, while the percent O/C decreased. This alteration might result from the degradation of ether bonds within lignin during the switchable solvent treatment. The elemental tests indicate a rise in the nitrogen content for the SSL sample. This may be attributed to the inclusion of minimal quantities of switchable solvent with lignin throughout the treatment process [61]. The SSL sample contained N, which is possibly from the DBU residue, therefore confirmed the low purity of the SSL sample.

**Figure 4:**
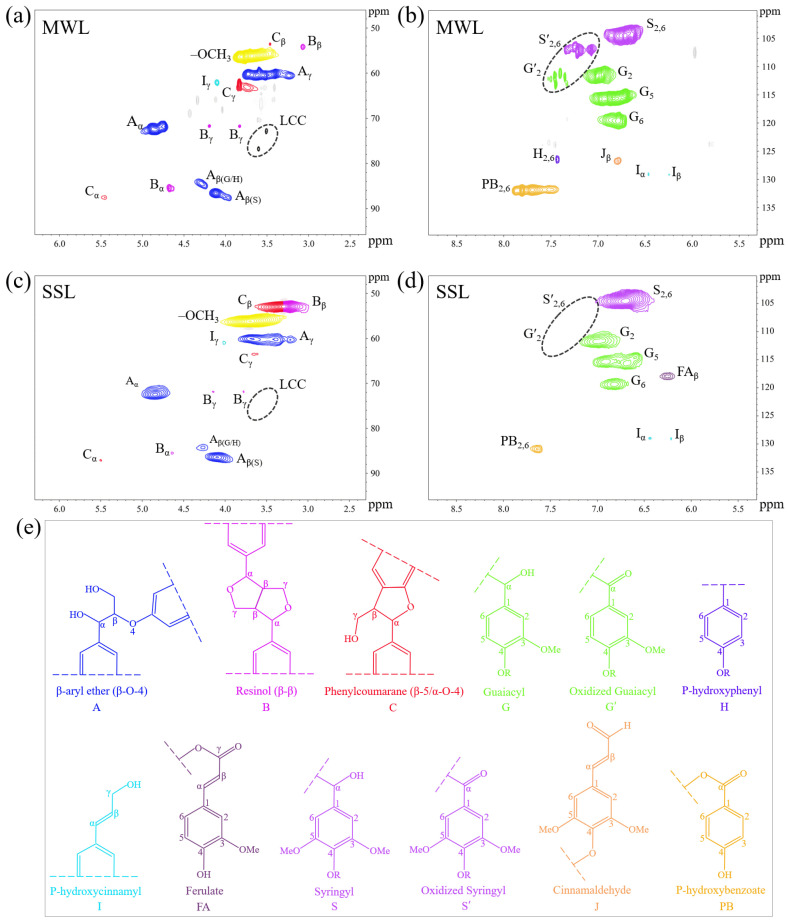
Two-dimensional HSQC spectra of MWL and SSL samples obtained from poplar wood: (**a**) side chain regions of MWL; (**b**) aromatic regions of MWL; (**c**) side chain regions of SSL; (**d**) aromatic regions of SSL; (**e**) main structures present in lignin samples: (A) β-O-4-aryl ether; (B) resinol; (C) phenylcoumaran; (G) guaiacyl; (G′) oxidized guaiacyl; (H) p-hydroxyphenyl; (I) p-hydroxycinnamoyl; (FA) ferulate; (S) syringyl; (S′) oxidized syringyl; (J) cinnamaldehyde; (PB) p-hydroxybenzoate.

A detailed structural analysis of the MWL and SSL samples was conducted using 2D HSQC NMR spectroscopy. Lignin is composed of two regions: the side chains, which detected at δ_C_/δ_H_ 50–90/2.5–6.0 ppm, and the aromatic rings, detected at δ_C_/δ_H_ 100–150/5.5–8.5 ppm [63]. The predominant subunits found in the side chain area are β-O-4 ethers, α-O-4 ethers, β-β resinols, β-5 phenyl coumarins, and α-alkoxy ethers [64], as shown in Figure 4a,c. The main lignin cross-peaks assigned to the 2D HSQC spectrum is detailed in Table S3. The –OCH_3_, being the most characteristic and prevalent in lignin, exhibits a pronounced signal at δ_C_/δ_H_ 56.3/3.69 ppm [65]. The primary subunits in the aromatic area are uncondensed and condensed G (G_2_, G_5_, and G_6_) along with uncondensed and condensed S (S_2,6_) [65,66], as shown in Figure 4b,d. The S unit signals at C_2,6_–H_2,6_ is observed at δ_C_/δ_H_ 103.9/6.69 ppm for S_2,6_ and δ_C_/δ_H_ 106.3/7.29 ppm for S′_2,6_. For the G units, four distinct peaks are identified: C_2_–H_2_ at δ_C_/δ_H_ 110.9/6.95 ppm (G_2_), C_2_–H_2_ at δ_C_/δ_H_ 111.4/7.51 ppm (G_2_′), C_5_–H_5_ at δ_C_/δ_H_ 114.9/6.75 ppm (G_5_), and C_6_–H_6_ at δ_C_/δ_H_ 118.9/6.80 ppm (G_6_). The disappearance of S′_2,6_ and G′_2_ cross-signal peaks, and the reduction in PB peaks in the SSL sample, may cause by the selective removal of oxidized S and G fragments during the switchable solvent treatment.

The distribution of interunit connections and the S/G ratio were analyzed through semi-quantitative analysis derived from 2D HSQC NMR spectrometer [38,39,67]. Table S4 indicates that the β-O-4 linkages content in the MWL sample was 57.69/100Ar, whereas the β-O-4 linkages content in the SSL sample, obtained by switchable solvent treatment, was 57.14/100Ar. Lignin with a greater number of β-O-4 linkages often exhibits elevated MW and more comprehensive structures [40]. The MWL sample yielded β-β linkages of 5.77/100Ar and β-5 linkages of 2.88/100Ar cross peaks. In contrast, the SSL sample exhibited β-β linkages of 5.97/100Ar and β-5 linkages of 1.19/100Ar. As a non-nucleophilic organic superbase, DBU has been proved to cleavage C–C bond in many organic reactions. In addition, in alkaline conditions it has been widely reported that the α-O-4 is extremely vulnerable and ease to cleavage, while the stability of β-O-4 will depend on the nucleophilicity of the chemicals added. Therefore, the investigation on the changes in β-O-4 and C–C was focused, to verify the function of DBU based solvents. It is very promising to illustrate that the content of β-O-4 in MWL and SSL is quite close, and a slight decrease in β-5 (Table S4) is noticed, which illustrated that lignin depolymerization does not follow the conventional alkaline treatment route.

The breaking of C–C or α-O-4 bonds during the extraction of SSL sample was well illustrated [57]. The C–C linkage content in the SSL sample reduced, while the –OCH_3_ content rose, and the fraction of –OCH_3_-rich S-type compounds increased. This indicates that HexOH impeded the polycondensation process following the disruption of the lignin macromolecular structure within the reaction system [51]. Changes in the S/G ratio can provide an understanding of the lignin structure modifications across different processing steps. Compared to the MWL sample, the S/G ratio of the SSL sample increased to 1.47. This increase could be indicative of the degradation or elimination of G-type lignin. This phenomenon indicates that the aryl ether or C–C bonds in lignin extracted using switchable solvents were either degraded or condensed [40]. Theoretically, a greater presence of S-type precursor units favors the creation of β-O-4 bonds [68].

Lignin is connected to carbohydrates through various chemical linkages, in addition to ether and C–C linkages. This makes it difficult to efficiently separate lignin from lignocellulosic biomass [69]. The phenyl glycoside bonds, benzyl ethers, and esters are constituting the primary forms lignin-carbohydrate complexes (LCC) in wood. Alkaline environment is capable of oxidative cleavage of LCC [70]. Additionally, additional signals within the aromatic regions were detected and attributed to the p-hydroxybenzoate substructures (PB), p-hydroxycinnamyl alcohol end groups (I), as well as cinnamaldehyde end groups (J). The C–H correlation peaks for I_α_, I_β_, and J_β_ were located at δ_C_/δ_H_ 128.4/6.44, 128.2/6.25, and 126.1/6.76 ppm, respectively, and the disappearance of the J_β_ cross-signal peak in the SSL sample. The PB are a distinct structural component of poplar lignin, and they can be distinguished by the C_2,6_–H_2,6_ correlated peaks at δ_C_/δ_H_ 131.2/7.64 ppm (PB_2,6_) [62]. The findings revealed a significantly higher concentration of PB_2,6_ in the MWL sample compared to the SSL sample. The inconsistency arises from the breakdown of ester bonds in the process of switchable solvent treatment, resulting in only a minimal presence of PB_2,6_ in the SSL sample [63,68]. Additionally, the SSL sample observed a strong signal of δ_H_/δ_C_ 117.98/6.25 ppm (FA_β_) belonging to ferulic acid (FA). In short, aside from changes in the PB_2,6_ and β-5 contents, β-O-4, β-β, and p-hydroxycinnamyl alcohol end groups within MWL sample, the SSL sample’s spectral data did not reveal any significant structural alterations. This phenomenon again indicated that the major linkages of lignin remain intact after treatment.

**Figure 5:**
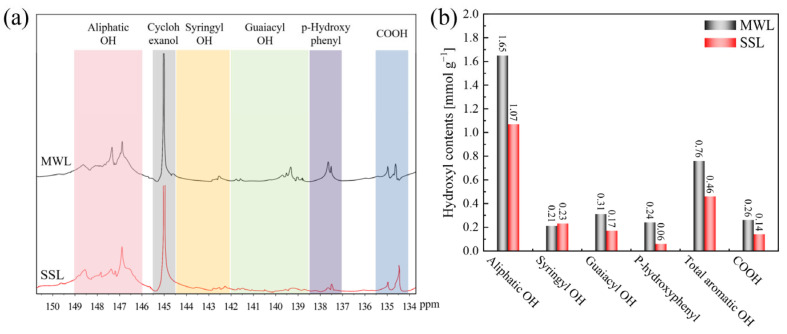
(**a**) Quantitative ^31^P NMR spectra of MWL and SSL; (**b**) Quantification of various –OH groups in lignin (mmol g^−1^).

The quantitative determination of –OH groups content can be achieved through ^31^P NMR spectroscopy, by utilizing methodologies described in the literature [54,71] to provide insight into the functional groups. Figure 5a shows the expanded –OH region (150–134 ppm) of the ^31^P NMR spectra of the MWL and SSL samples. Figure 5b illustrates that the switchable solvent treatment led to a 9.5% rise in the phenolic content of the S-type lignin, contrasted with the MWL sample, while the G-type and H-type phenolic contents decreased by 45.2% and 75.0%, respectively. The reduction in the aliphatic –OH content (1.07 mmol g^−1^) observed in the SSL sample can be ascribed to the dehydration of aliphatic –OH groups subjected to severe hydrothermal treatment. Among the phenolic hydroxyl groups, the rise in the proportion of butyl hydroxyl groups provided further evidence of the substantial presence of β-O-4 linkages connected with S-type units in the MWL. Within switchable solvent systems, lignin tends to undergo side chain scission during degradation and extraction, causing a decline in phenolic hydroxyl content [51]. The reduction in the total aromatic hydroxyl content (0.46 mmol g^−1^) observed in the SSL sample was influenced by the minimal cleavage of ether linkages caused by the switchable solvent. This, consequently, may limit the formation of hydroxyl groups [72]. Furthermore, the decrease in the quantity of phenolic –OH groups can be accounted for by the phenomenon of lignin’s self-polymerization, which occurs through the condensation reactions involving phenolic –OH functional groups [73].

**Figure 6:**
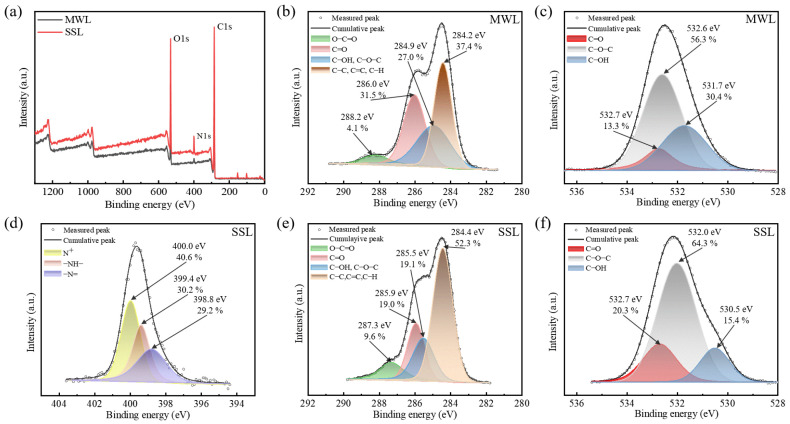
(**a**) XPS analysis of MWL and SSL samples; (**b**) C1s scan of MWL; (**c**) O1s scan of MWL; (**d**) N1s scan of SSL; (**e**) C1s scan of SSL; and (**f**) O1s scan of SSL.

Figure 6 displays the XPS survey scan spectra for the MWL and SSL samples. The spectra feature two prominent peaks at C1s (284.4 eV) and O1s (532.1 eV). A small amount of N1s (398.6 eV) is detected in the SSL sample. The C1s, O1s, and N1s core-level spectra from the samples were decomposed into three peak components for analysis. The comparative mass concentrations of the various bonds in the MWL and SSL samples are shown in Figure 6b–f. Compared to MWL, the SSL sample showed that the C–H/C–C/C=C ratio increased by 14.9%; C–OH/C–N/C–O–C increased by 7.9% as a result of oxidation; C=O decreased by 12.5%; and carboxylic O–C=O increased by 5.5%. This occurrence can be accounted for by the transformation of carbonyl groups into carboxylic acid groups throughout the oxidation process [63]. The SSL sample also showed that C=O and C–O–C increased by 7.0% and 8.0%, respectively. Conversely, the C–OH content decreased by 15.0%. The reduction in aliphatic or phenolic hydroxyl groups might be due to their transformation into carboxylic C=O groups. Figure 6d reveals that the curve deconvolution results in the presence of three peaks attributed to –N= (398.8 eV), –NH– (399.4 eV), and N^+^ (400.0 eV). The percentages of –N=, –NH–, and N^+^ were of 29.2%, 30.2%, and 40.6%, respectively. The source of N in the SSL sample may attribute to the residence of switchable solvent fragments, namely DBU–HexOH. And this can be confirmed in Figure S2, where flaw typical DBU–HexOH signal can be identified from 2D HSQC SSL sample.

### 3.3. Proposed Mechanism of Switchable Solvent Lignin Extraction

**Figure 7:**
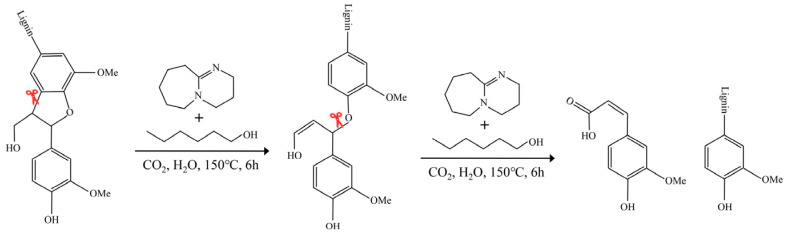
Possible mechanism for the lignin extraction reaction using the switchable solvent.

Lignin can be selectivity extracted from lignocellulose by using the proposed switchable solvent. Based on the research results and the literature [74,75], a mechanism for SSL extraction was proposed as shown in Figure 7. Switchable solvent with the presence of CO_2_ can destroy the hydrogen bonds between lignin, hemicellulose, and cellulose molecules, thereby facilitating the liberation lignin from lignocellulosic biomass. Notably, it possesses a substantial ability to alter the hydrogen-bonding structure, simultaneously enabling the establishment of strong hydrogen bonds with lignin, hence facilitating lignin extraction [31]. Different from other ways of extracting lignin, the switchable solvent treatment process focuses on the breaking of C–C bond (β-5) and some specific ether bond (α-O-4) in lignin, as shown in Figure 7. The switchable solvent facilitated the breaking of partial α-O-4 and C–C bonds in the lignin sample. Nevertheless, the vast β-O-4 linkages remained mostly intact, resulting in the release of the SSL sample with an intact structure and with less condensed fragments. At the end of the reaction, the gas CO_2_ was removed from the switchable solvent-lignin mixture to reduce the solubility of lignin. This not only facilitated the separation process but also inhibited the potential condensation reactions of lignin segments. In summary, the switchable solvent system within this research effectively extracted structurally intact lignin from poplar biomass, with a high β-O-4 bonds, high MW, and improved uniformity, which is conducive to its high value in bio-based functional materials.

### 3.4. Lignin Nanoparticles

**Figure 8:**
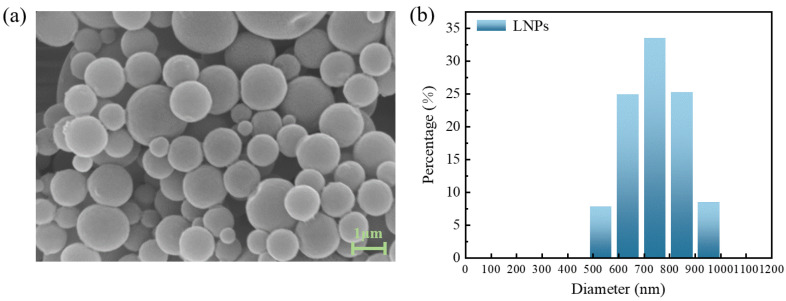
Characterizations of LNP: (**a**) SEM images of LNPs; (**b**) Particle size distributions of LNPs.

The SSL was effectively transformed into LNPs using the GVL solvent exchange self-assembly technique. Throughout this transformation, the self-assembly process is governed by a mechanism that orchestrates the formation of nanoparticles through the exploitation of interactions such as hydrophobic and hydrophilic forces, hydrogen bonding, and π–π interactions [76]. Its microstructure is shown in Figure 8a. LNPs showed a uniform spherical structure and smooth surface. The particle size distribution of LNP is shown in Figure 8b. The size range of lignin spherical particles prepared by SSL self-assembly method is between 531 and 955 nm, which is consistent with the conclusion observed in SEM images. The Zeta potential of LNPs in water system was −25.67 ± 0.43 mV. The surfaces of the LNPs carried a negative charge, which arises from the deprotonation of phenolic hydroxyl and carboxylic acid functionalities present in the lignin macromolecule [77]. The negative surface charge of the LNPs contributes to their electrostatic stabilization, thereby inhibiting their tendency to aggregate. The results showed that LNPs with regular shape and particle size distribution were successfully prepared by using lignin prepared by switchable solvent treatment.

## 4. Conclusions

A switchable solvent composed of DBU, hexanol, DI, and bulging gas CO_2_ can efficiently selectively separate and extract lignin from poplar wood, and the extracted switchable solvent lignin had the characteristics of higher MW and higher thermal stability, compared to MWL. The findings demonstrated that the switchable solvent treatment method has a significant positive effect on the properties and structure of the extracted lignin. It can break the C–C and α-O-4 bonds of lignin macromolecules. The switchable solvent treatment method can accurately control the extraction process. The uniformity and stability of the lignin product can be enhanced by optimizing the process parameters. SSL sample was further prepared into LNPs with regular shape and uniform particle size distribution. An efficient and practical approach for extraction and evaluation of lignin was presented in this work, which could be applied in green chemistry.

## Data Availability

Data is contained within the article or Supplementary Materials.

## Supplementary Materials

The following supporting information can be downloaded at: https://www.mdpi.com/article/10.3390/polym16243560/s1, Figure S1: Response surfaces for lignin removal in poplar wood using switchable solvent; Table S1. Molecular weight and molecular dispersity indices of the recovered lignin; Table S2. Assignment of FT-IR of lignin samples; Table S3. Assignments of ^13^C–^1^H correlation signals in the HSQC NMR spectra of MWL and SSL samples; Table S4. Quantification of lignin fractions by quantitative 2D-HSQC NMR spectroscopy; Figure S2. Two-dimensional HSQC spectra of switchable solvent and SSL sample.
