# Supplementary material for: Switchable Solvent for Separation and Extraction of Lignin from Lignocellulose Biomass: An Investigation of Chemical Structure and Molecular Weight
Source: Polymers (Basel). 2024 Dec 20;16(24):3560. doi: 10.3390/polym16243560 (PMC11679162; doi:10.3390/polym16243560)
Supplement: Supplementary file 1 [file polymers-16-03560-s001.zip › polymers-3361137-supplementary.pdf]

polymers

MDPI

Supplementary Materials

Switchable solvent for separation and extraction of lignin from lignocellulose biomass: an investigation on chemical structure and molecular weight

Debao Li [1], Letian Qi [1,*], Magdi E. Gibril [1,2], Yu Xue [1], Guihua Yang [1,*], Mengru Yang [1], Yujie Gu [1] and Jiachuan Chen [1]

1. Methods

1.1. Preparation of MWL

The preparation of MWL was carried out using the procedure described in the existing literature [1]. A solution of benzene and ethanol with a volume ratio of 2:1 was used to extract 80~40 mesh of poplar powder, after which the desiccated material was subjected to a planetary ball mill (Pulverisette 5, FRITSCH, Germany) for 2 days at 300 rpm. The sample underwent extraction with a mixture of 1,4-dioxane and DI at a ratio of 96:4 (v/v). Then, centrifugation was employed to separate the insoluble components. The 1,4-dioxane was then eliminated using rotary evaporation, after which the sample was freeze-dried for 24 h to collect MWL samples.

1.2 Semi-quantitative analysis of substructures

2D HSQC NMR was utilized for semi-quantitative analysis of the MWL and SSL samples, and relative content of the substructures was described as content per 100 aromatic units [38,39,67]. To start, the collective aromatic regions are delineated as follows:

$IC9\ units = 0.5I(S_{2,6}) + I(G_2)$;

The total aromatic areas are defined by the integrals: $I(S_{2,6})$ for $S_{2,6}$, $I(G_2)$ for G_2, and I(C9) for the aromatic ring. Using the internal standard I(C9), the percentages of I(β-O-4), I(β-β), and I(β-5) can be calculated with the following equation:

$I(\beta\text{-O-4})(\%) = (IA_\alpha)/IC9\times100\%$;

$I(\beta\text{-}\beta)(\%) = (IB_\alpha)/IC9\times100\%$;

$I(\beta\text{-5})(\%) = (IC_\alpha)/IC9\times100\%$;

Where I(β-O-4), I(β-β), and I(β-5) are the integral value of the α-position of A_α, B_α, and C_α, ensure the integration occurs within a consistent contour level.

2. Figures and Tables

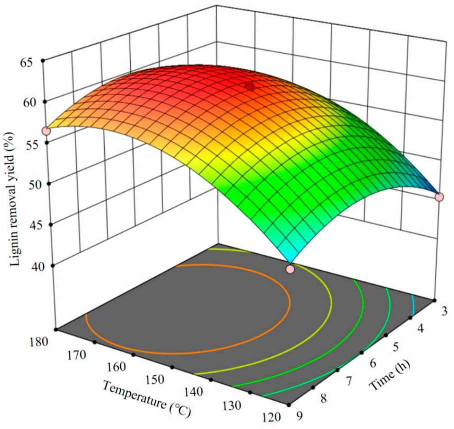

Figure S1. Response surfaces for lignin removal in poplar wood using switchable solvent.

Table S1. Molecular weight and molecular dispersity indices of the recovered lignin.

Sample	M_n (g/mol)	M_w (g/mol)	Đ
ChCl-Lac lignin [49]	846	2643	3.12
LigAc [50]	3075	5963	1.98
MWL	3400 ± 90	6400 ± 127	1.88 ± 0.02
SSL	4400 ± 66	8000 ± 44	1.82 ± 0.02

Table S2. Assignment of FT-IR analysis of lignin samples.

Wave-numbers(cm^{-1})	Assignment (Bond)
3414	O–H stretching vibration
2937	C–H stretching vibration in methyl
2862	C–H vibration of the $-OCH_3$ groups
1711	C=O stretching vibration
1508	Aromatic ring skeleton vibration
1459	C–H bending from methyl or methylene groups
1325	Syringyl ring breathing with C–O stretching vibrations
1268	Guaiacyl ring breathing with C–O stretch in lignin, C–O linkage in guaiacyl aromatic methoxyl groups
1225	C–O(H) stretching of phenolic OH and ether
1123	C–H stretching vibration of syringyl units
1035	C–H bending vibration of guaiacyl units
832	Aromatic C–H out of plane bending of Syringyl units

Table S3. Assignments of ^{13}C–^{1}H correlation signals in the HSQC NMR spectra of MWL and SSL samples.

Labels	δC/δH (ppm)	Assignment
C_β	53.8/3.46	C_β–H_β in phenylcoumaran substructures (C)
B_β	53.5/3.07	C_β–H_β in resinol substructures (B)
$-OCH_3$	55.6/3.73	C–H in methoxyls
A_γ	59.5-59.7/3.34-3.79	C_γ–H_γ in β-O-4′ substructures (A)
A'_γ	63.2/4.33-4.49	C_γ–H_γ in γ-acylated β-O-4′ substructures (A′)
C_γ	62.7/3.73	C_γ–H_γ in phenylcoumaran substructures (C)

I_{γ}	62.1/4.11	C_{γ}–H_{γ} in p-hydroxycinnamyl alcohol end groups (I)
B_{γ}	71.6/3.82 and 4.18	C_{γ}–H_{γ} in resinol substructures (B)
A_{α}	72.2/4.86	C_{α}–H_{α} in β-O-4′ substructures (A)
$A_{\beta(G/H)}$	84.4/4.29	C_{β}–H_{β} in β-O-4′ substructures linked to G/H units (A)
B_{α}	85.5/4.65	C_{α}–H_{α} in resinol substructures (B)
$A_{\beta(S)}$	86.6/4.12	C_{β}–H_{β} in β-O-4′ substructures linked to S units (A)
$S_{2,6}$	104.4/6.71	$C_{2,6}$–$H_{2,6}$ in etherified syringyl units (S)
$S'_{2,6}$	107.2/7.23 and 7.07	$C_{2,6}$–$H_{2,6}$ in oxidized (C_{α}=O) syringyl units (S′)
G_{2}	111.5/6.98	C_{2}–H_{2} in guaiacyl units (G)
G_{2}'	111.4/7.40	$C_{2,6}$–$H_{2,6}$ in oxidized (C_{α}=O) guaiacyl units (G′)
G_{5}	115.5/6.78	C_{5}–H_{5} in guaiacyl units (G)
G_{6}	119.5/6.80	C_{6}–H_{6} in guaiacyl units (G)
I_{β}	128.8/6.25	C_{β}–H_{β} in p-hydroxycinnamyl alcohol end groups (I)
I_{α}	129.1/6.44	C_{α}–H_{α} in p-hydroxycinnamyl alcohol end groups (I)
$PB_{2,6}$	131.2/7.67	$C_{2,6}$–$H_{2,6}$ in p-hydroxybenzoate substructures (PB)
J_{β}	126.6/6.76	C_{β}–H_{β} in cinnamaldehyde end groups (J)
$H_{2,6}$	127.9/7.44	$C_{2,6}$–$H_{2,6}$ in p-hydroxyphenyl units (H)
FA_{β}	117.9/6.25	C_{β}-H_{β} in ferulates (FA)

Table S4. Quantification of lignin fractions by quantitative 2D-HSQC NMR spectroscopy.

**Sample**	**β-O-4** [a]	**β-β** [a]	**β-5** [a]	**S/G** [b]
MWL	57.69	5.77	2.88	0.93
SSL	57.14	5.95	1.19	1.47

* a Results expressed per 100Ar based on quantitative 2D HSQC spectra.

b The S/G ratio of lignin was determined using $C_{2,6}$–$H_{2,6}$ correlations from S units and the C_{2}–H_{2} correlation from G units in the aromatic region [39]. The S/G ratio was computed using the formula: $S/G = 0.5I(S_{2,6})/I(G_{2})$.

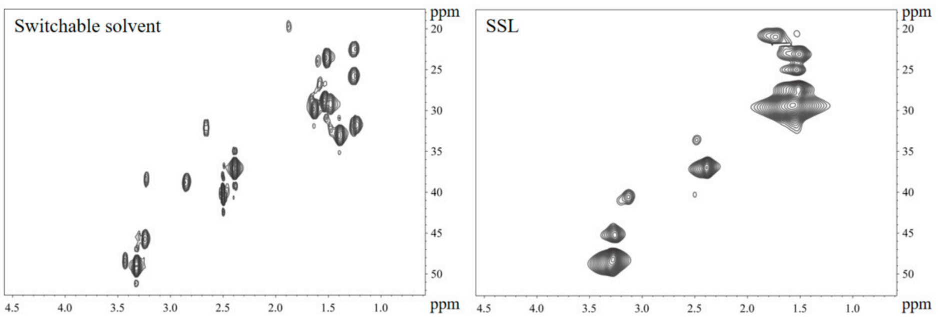

Figure S2. Two-dimensional HSQC spectra of switchable solvent and SSL samples.

Disclaimer/Publisher's Note: The statements, opinions and data contained in all publications are solely those of the individual author(s) and contributor(s) and not of MDPI and/or the editor(s). MDPI and/or the editor(s) disclaim responsibility for any injury to people or property resulting from any ideas, methods, instructions or products referred to in the content.
